# Investigating the Association of Genetic Markers, Previously Identified by Genome‐Wide Association Studies, With ACL Rupture in Multiple Cohorts

**DOI:** 10.1002/jor.70282

**Published:** 2026-09-21

**Authors:** Caryn Phipson, Mary‐Jessica N. Laguette, Paweł Cieszczyk, Krzysztof Ficek, Charlotte K Häger, Eva‐Lena Stattin, Kjell G Nilsson, Julian A Feller, Oren Tirosh, Alison V September, Malcolm Collins

**Affiliations:** ^1^ Health through Physical Activity, Lifestyle and Sport Research Center (HPALS), Division of Physiological Sciences, Department of Human Biology University of Cape Town Cape Town South Africa; ^2^ Faculty of Physical Education Gdańsk University of Physical Education and Sport Gdańsk Poland; ^3^ Faculty of Physiotherapy Jerzy Kukuczka Academy of Physical Education in Katowice Katowice Poland; ^4^ Department of Community Medicine and Rehabilitation Umeå University Umeå Sweden; ^5^ Department of Immunology Genetics and Pathology, Uppsala University Uppsala Sweden; ^6^ Department of Diagnostics and Intervention Umeå University Umeå Sweden; ^7^ OrthoSport Victoria Research Unit Epworth Healthcare Victoria Australia; ^8^ RMIT University Melbourne Australia

**Keywords:** anterior cruciate ligament, genetic markers, genetic testing, genome‐wide association study, polymorphism

## Abstract

Several direct‐to‐consumer genetic tests claim to predict susceptibility to anterior cruciate ligament (ACL) rupture. Although not independently replicated, polymorphisms identified in a genome‐wide association study—rs144051132 (T > A), rs186727643 (C > T), and/or rs188099931 (A > G)—have been incorporated into commercial tests to estimate cruciate ligament injury risk. This study aimed to evaluate whether these polymorphisms are associated with ACL rupture across multiple populations. We genotyped 906 clinically diagnosed ACL rupture cases, including 498 non‐contact mechanism of injury ruptures (ACL‐NC), and 679 controls (CON) from four European ancestry cohorts and a South African mixed‐ancestry cohort. No significant differences were observed in genotype distributions between cases and controls in either the European cohorts (rs144051132 CON: 99.3% TT, 0.7% TA; ACL: 99.0% TT, 1.0% TA; rs186727643 CON: 100.0% CC; ACL: 99.9% CC, 0.1% CT or rs188099931 CON: 99.8% AA, 0.2% AG; ACL: 99.4% AA, 0.6% AG) or the mixed‐ancestry cohort (rs144051132 CON: 99.0% TT, 1.0% TA; ACL: 97.9% TT, 2.1% TA; rs186727643 CON: 97.1% CC, 2.9% CT; ACL: 94.8% CC, 5.2% CT or rs188099931 CON: 99.0% AA, 1.0% AG; ACL: 100.0% AA). These polymorphisms were not associated with ACL rupture in the studied populations. The study was adequately powered to detect the larger effects previously reported but underpowered to detect the smaller effect reported for rs188099931. These results, as well as the rarity of the minor alleles, do not support the clinical validity of these polymorphisms as markers of ACL rupture risk.

## 1 Introduction

Anterior cruciate ligament (ACL) rupture is a multifactorial injury with a substantial genetic component. Heritability estimates of up to 69% have been reported in a large Swedish twin study, indicating that genetic factors play a key role in predisposing individuals to ACL rupture and contribute significantly to the observed inter‐individual variation in injury susceptibility [1]. Although our understanding of the genetic contribution to injury risk has increased markedly over the past two decades, alongside rapid advances in genomic technologies, current knowledge remains relatively limited [2].

Nevertheless, several direct‐to‐consumer (DTC) genetic testing companies market tests that claim to predict susceptibility to various musculoskeletal soft tissue injuries, including ACL rupture [3, 4]. Most companies have incorporated genetic markers identified using candidate‐gene approaches into their commercial tests for assessing ACL injury risk. More recently, genetic markers identified through genome‐wide association studies (GWAS) have also been incorporated into commercial testing panels [3]. This GWAS‐based approach is generally regarded as more robust and scientifically reliable than the traditional candidate‐gene approach for identifying genetic polymorphisms associated with complex phenotypes [3, 5].

Significant independent associations between the polymorphisms rs144051132 (T > A), located in a regulatory region of the *INHBA* gene; rs186727643 (C > T), an intronic polymorphism in *LOC101928387*; and rs188099931 (A > G), an intergenic polymorphism, and ACL and/or PCL injury risk were reported in a GWAS analyzing data from the Kaiser Permanente Research Bank (KPRB) and the UK Biobank [5]. Specifically, the minor rs144051132 A, rs186727643 T, and rs188099931 G alleles were associated with an increased risk of injury. However, these findings have not yet been replicated in independent, well‐characterized populations. Furthermore, the biological significance of these markers remains unclear.

The primary aim of this study was therefore to determine whether the minor rs144051132 A, rs186727643 T, and rs188099931 G alleles are associated with ACL rupture across multiple international cohorts. The secondary aim was to independently confirm the clinical validity of using these polymorphisms as markers of ACL rupture risk.

## 2 Methods

### 2.1 Study Design and Setting

A case‐control genetic association study was designed, comprising of cases clinically diagnosed with ACL ruptures (ACL), a subset of cases who sustained an ACL rupture via a non‐contact mechanism (ACL‐NC), and controls (CON). The study adhered to the guidelines outlined in the STREGA (Strengthening the Reporting of Genetic Association studies) report, an extension of the STROBE (Strengthening the Reporting of Observational Studies in Epidemiology) statement [6]. Ethical approval was obtained by the Human Research Ethics Committee of the Faculty of Health Sciences at the University of Cape Town (UCT) (HREC 176/2024) as well as the Epworth Hospital in Victoria, Australia (HREC approval: 57012), the Bioethics Committee for Clinical Research, Regional Medical Chamber in Gdansk, Poland (KB‐8/16) and the Regional Ethical Review Board in Umeå, Sweden (dnr. 2011‐200‐31 M). All laboratory work for this study was performed at UCT's Health through Physical Activity, Lifestyle and Sport (HPALS) Research Center laboratories at the Sports Science Institute of South Africa (Newlands, Cape Town).

### 2.2 Participants

Informed consent, data and DNA was obtained from four cohorts comprising participants of self‐reported European ancestry from Australia, Poland, South Africa and Sweden, and one South African cohort of self‐reported mixed ancestery as previously described [7, 8, 9, 10, 11, 12, 13]. Briefly, prior to participation, participants were required to sign a written informed consent form, in accordance with the Declaration of Helsinki, as well as complete a questionnaire regarding personal particulars, physical and sporting activity, and personal and family medical/injury history. All ACL ruptures were clinically diagnosed based on physical examination and confirmed by either magnetic resonance imaging (MRI), arthroscopy, or during surgery. Controls were required to have no previous history of tendon or ligament injury. All participants participated in regular sporting activities, primarily at a recreational level.

The Australian cohort comprised of 412 participants (ACL = 330, ACL‐NC = 152, CON = 82) recruited between 2006 and 2018 in Melbourne, Australia [7, 12]. The Polish cohort comprised of 289 participants (ACL = 147, ACL‐NC = 56, CON = 142) recruited between 2008 and 2018 in Beiruń, Poland [9]. The South African cohort of self‐reported European ancestry comprised of 471 participants (ACL = 242, ACL‐NC = 161, CON = 229) recruited between June 2006 and February 2016 from the greater Cape Town region [11]. The Swedish cohort comprised of 211 participants (ACL = 95, ACL‐NC = 79, CON = 116) recruited between 2011 and 2013 across the two most northern regions of Sweden [8].

For this study, South African mixed ancestry refers to a distinct population group based in the Western Cape that has an amalgamation of African, European, and Asian ancestry [14]. The cohort comprised of 202 South African participants of mixed ancestry (ACL = 97, ACL‐NC = 50, CON = 105) who were previously recruited between January 2012 and May 2016 from the greater Cape Town region [13].

### 2.3 DNA Extraction and Genotyping

As this study made use of existing DNA samples, all sample collections and DNA extractions had been performed prior to the study's commencement, as previously described [7, 8, 9, 10, 11, 12, 13]. Genotyping was conducted using the TaqMan assay protocol, a real‐time PCR‐based assay designed specifically for genotyping single nucleotide polymorphisms (SNPs). Custom‐designed fluorescence‐based Taqman Genotyping assays (ThermoFisher Applied Biosystems, Foster City, USA) were used to genotype the DNA samples for the rs144051132 (T > A), rs186727643 (C > T), and rs188099931 (A > G) polymorphisms according to the manufacturer's instructions. Oligonucleotide probes, synthesized to match the sequences of each of the three polymorphisms, were used as positive test controls to verify the accuracy and reliability of the genotyping results by providing a known positive signal for each genotype (Supporting Information Table S1 and Supporting Information Figures S1 to S3). Quantitative real‐time PCR was performed using the QuantStudio 3 PCR machine (ThermoFisher Applied Biosystems, Foster City, USA). Genotype calling and data analysis was performed using the ThermoFisher Cloud Genotyping Suite SDS3.2 software (https://apps.thermofisher.com/apps/spa/).

Sanger sequencing was performed in a subset of samples (*n* = 105) from each cohort to confirm the genotyping results, including all samples with a rare genotype (Inqaba Biotechnical Industries, Pretoria, South Africa) (Supporting Information Figures S4 to S6). Unipro UGENE software (version 51.0) was used to analyze the sequencing data and generate chromatogram plots to visualize the DNA sequences [15].

### 2.4 Statistical Analyzes

Power calculations to determine sample sizes were performed using the University of Michigan's web interface GAS Power Calculator. Assuming an injury‐associated allele frequency of 1.1% for rs144051132, a sample of 601 cases and controls would be sufficient to detect an odds ratio of 2.9 with 81% power at a significance level of 5%. Similarly, assuming an injury‐associated genotype frequency of 0.2% for rs186727643, a sample of 1202 cases and controls would be sufficient to detect an odds ratio of 4.5 with 80% power at a significance level of 5%. However, a substantially larger sample size of 4800 cases and controls would be required to detect an odds ratio of 2.6 for rs188099931 (https://csg.sph.umich.edu/abecasis/cats/gas_power_calculator/) [5].

RStudio (version R 4.6.0), IBM SPSS Statistics Software (version 30.0.0) and GraphPad Prism (version 10.4.2) were used for all statistical analyzes, with significance set at *p* < 0.05. Age, height, weight and BMI were determined at the time of initial injury and/or time of recruitment for the ACL participants, and at the time of recruitment for the CON participants. The Shapiro‐Wilk test for normality was used to examine the distribution of continuous data. Parametric data were presented as mean ± standard deviation and non‐parametric data were presented as median and interquartile ranges (Q1; Q3). Parametric two‐group comparisons were analyzed using the independent samples t‐test, with a Mann Whitney‐U test used for non‐parametric two‐group comparisons. Potential confounding variables were included in the descriptive analyzes where necessary and significance values adjusted accordingly, using the Quade non‐parametric ANCOVA test. Categorical data were presented as frequencies and analyzed using the Pearson Chi‐Squared test or Fisher's Exact test. The R package SNPassoc was also used to analyse the differences in genotype frequencies of each polymorphism amongst the groups [16]. While the codominant model was estimable for the three variants, the absence of minor allele homozygotes and the low frequency of heterozygous genotypes precluded reliable estimation of the dominant, recessive, overdominant and log‐additive inheritance models. Although the log‐additive model was also estimable for the genotype scores, it produced the same results as the codominant model because only two genotype categories were observed. Therefore, only the codominant model was reported. The combined European analysis was covariate‐adjusted for country of recruitment to reduce the effects of population stratification, while the South African mixed‐ancestry analysis was covariate‐adjusted for differences in participant characteristics between the groups.

### 2.5 Bioinformatics Analyzes

Scores indicating the functional and/or pathogenic effect of each polymorphism were determined using multiple bioinformatics tools, including the Ensembl Variant Effect Predictor (VEP) (https://www.ensembl.org/Homo_sapiens/Tools/VEP, accessed 15 April 2025), the ClinVar database (https://www.ncbi.nlm.nih.gov/clinvar/, accessed 15 April 2025), as well as Combined Annotation Dependent Depletion (CADD) (https://www.ensembl.org/index.html, accessed 15 April 2025), SIFT (https://sift.bii.a-star.edu.sg/www/SIFT_dbSNP.html, accessed 15 April 2025), and FATHMM scores (https://fathmm.biocompute.org.uk/fathmmMKL.html, accessed 15 April 2025). The potential functional effects of sequence variation on RNA secondary structure were determined using S‐fold software (https://sfold.wadsworth.org/cgi-bin/srna.pl).

## 3 Results

### 3.1 General Characteristics

In the combined analysis of the four cohorts of self‐reported European ancestry, there were no significant differences in sex and height between the CON and ACL groups, as well as between the CON group and ACL‐NC sub‐group (Table 1). At the time of injury, the ACL group (26.0 [20.0; 34.0] years, *n* = 715, *p* = 0.001) and ACL‐NC sub‐group (26.0 [20.0; 34.0] years, *n* = 448, *p* = 0.015) were significantly older than the CON group at recruitment (25.0 [21.0; 39.0] years, *n* = 567). After covarying for age, the ACL group and ACL‐NC sub‐group were significantly heavier, with a corresponding greater BMI, than the CON group at the time of injury and at the time of recruitment (Table 1). The general characteristics of each individual cohort are summarized in Supporting Information Tables S2 to S5.

**Table 1:** Participant characteristics of the apparently healthy control (CON) and anterior cruciate ligament (ACL) groups, as well as between the CON group and a subset of the ACL group (ACL‐NC), comprising those who sustained ACL ruptures via a non‐contact mechanism, of the combined cohorts (Australian, Polish, South African, and Swedish) of self‐reported European ancestry.

	CON	ACL	*p*‐value	ACL‐NC	*p*‐value
	*n* = 574	*n* = 809		*n* = 448	
Age (years)	25.0 (21.0; 39.0)^a^	26.0 (20.0; 34.0)^b^	**0.001**	26.0 (20.0; 34.0)^b^	**0.014**
Sex (% males)	62.9	63.2	0.954	62.3	0.845
Height (cm)	175.0 (169.0; 182.0)	177.0 (169.0; 183.0)	0.463	177.0 (170.0; 183.0)	0.499
Weight (kg)	74.0 (65.0; 81.6)^a^	77.0 (66.1; 86.1)^b^	**< 0.001** c	76.2 (65.0; 81.6)^b^	**0.003** c
		79.0 (68.0; 90.0)^a^	**< 0.001**	78.0 (67.9; 88.3)^a^	**< 0.001**
BMI (kg/m^2^)	23.5 (21.9; 25.4)^a^	24.4 (22.5; 26.9)^b^	**< 0.001** c	24.4 (22.2; 26.9)^b^	**< 0.001** c
		24.9 (22.8; 27.7)^a^	**< 0.001**	24.8 (22.7; 27.5)^a^	**< 0.001**

*Note:* Except for sex, which is expressed as a percentage, the remaining continuous variables are expressed as average ± standard deviation or median and interquartile range (Q1; Q3) for parametric and non‐parametric data, respectively. *p*‐values in bold indicate significance (*p* < 0.05). Age (*n*): CON = 567; ACL = 715; ACL‐NC = 441. Sex (*n*): CON = 574; ACL = 744; ACL‐NC = 448. Height (*n*): CON = 558; ACL = 710; ACL‐NC = 435. Weight (*n*): CON = 560; ACL ^1^ = 449; ACL ^2^ = 558; ACL‐NC ^1^ = 286; ACL‐NC ^2^ = 369. BMI (*n*): CON = 555; ACL ^1^ = 442; ACL ^2^ = 556; ACL‐NC ^1^ = 282; ACL‐NC ^2^ = 368.

Abbreviations: BMI, body mass index; cm, centimetres; kg, kilograms; m, meters.

^a^ Self‐reported at time of recruitment.

^b^ Self‐reported at time of initial ACL injury.

^c^ Adjusted for age.

At the time of recruitment, there were no significant differences in age, sex, height and weight between the South African mixed ancestry CON and ACL groups, as well as the CON group and ACL‐NC sub‐group (Table 2). Similarly, there were no significant differences in weight between either the ACL group or ACL‐NC sub‐group at time of injury and CON group at recruitment. However, both the ACL group (24.0 [19,0; 30,0] kg, *n* = 91, *p* = 0.003) and ACL‐NC sub‐group (23.0 [17.0; 29.3] kg, *n* = 50, *p* = 0.010) were significantly younger at the time of injury compared to the CON group at recruitment (27.0 [22.3; 30.0] kg, *n* = 104). While there were no significant differences in BMI between the CON and ACL groups, the ACL‐NC sub‐group at both the time of injury (23.9 [21.2; 27.2] kg/m^2^, *n* = 47, *p* = 0.026) and time of recruitment (23.4 [21.2; 27.1] kg/m^2^, *n* = 48, *p* = 0.010) had a significantly lower BMI than the CON group (25.8 [23.2; 27.8] kg/m^2^, *n* = 99) (Table 2).

**Table 2:** Participant characteristics of the apparently healthy control (CON) and anterior cruciate ligament (ACL) groups, as well as between the CON group and a subset of the ACL group (ACL‐NC), comprising those who sustained ACL ruptures via a non‐contact mechanism, of the South African mixed ancestry cohort.

	CON	ACL	*p*‐value	ACL‐NC	*p*‐value
	*n* = 105	*n* = 97		*n* = 50	
Age (years)	27.0 (22.3; 30.0)^a^	24.0 (19,0; 30,0)^b^	**0.003**	23.0 (17.0; 29.3)^b^	**0.010**
		26.0 (20.0; 32.0)^a^	0.561	25.0 (19.5; 31.0)^a^	0.254
Sex (% males)	81.9	82.5	1.000	88.0	0.484
Height (cm)	172.2 ± 8.2	173.5 ± 8.7	0.283	174.3 ± 8.8	0.180
Weight (kg)	76.5 (67.0; 87.0)^a^	76.5 (66.5; 87.9)^b^	0.883	73.0 (65.5; 83.5)^b^	0.306
		75.5 (65.8; 89.3)^a^	0.906	71.5 (64.3; 81.7)^a^	0.100
BMI (kg/m^2^)	25.8 (23.3; 27.8)^a^	25.2 (21.9; 28.4)^b^	0.718	23.9 (21.2; 27.2)^b^	**0.026**
		25.2 (22.0; 28.2)^a^	0.762	23.4 (21.2; 27.1)^a^	**0.010**

*Note:* Except for sex, which is expressed as a percentage, the remaining continuous variables are expressed as average ± standard deviation or median and interquartile range (Q1; Q3) for parametric and non‐parametric data, respectively. p‐values in bold indicate significance (*p* < 0.05). Age (*n*): CON = 104; ACL ^1^ = 93; ACL ^2^ = 91; ACL‐NC ^1^ = 50; ACL‐NC ^2^ = 50. Sex (*n*): CON = 105; ACL = 97; ACL‐NC = 50. Height (*n*): CON = 100; ACL = 86; ACL‐NC = 47. Weight (*n*): CON = 99; ACL ^1^ = 90; ACL ^2^ = 88; ACL‐NC ^1^ = 50; ACL‐NC ^2^ = 49. BMI (*n*): CON = 99; ACL ^1^ = 85; ACL ^2^ = 83; ACL‐NC ^1^ = 48; ACL‐NC ^2^ = 47.

Abbreviations: BMI, body mass index; cm, centimetres; kg, kilograms; m, meters.

^a^ Self‐reported at time of recruitment.

^b^ Self‐reported at time of initial ACL injury.

There were no differences in the general characteristics between the genotype groups of the three polymorphisms (Supporting Information Tables S6 to S11).

### 3.2 Associations Between rs144051132 (T ≫ A), rs186727643 (C ≫ T) and rs188099931 (A ≫ G), and ACL Rupture Risk

There were no significant differences in the rs144051132 (T > A), rs186727643 (C > T) or rs188099931 (A > G) genotype or allele distributions between the CON and ACL groups, or between the CON group and ACL‐NC sub‐group, when the combined cohorts of self‐reported European ancestry was analyzed (Table 3). These differences remained non‐significant after covarying for country of recruitment. Similarly, there were no significant differences in the relative genotype distibutions of any of the three polymophisms between the groups when the cohorts were analyzed individually (Supporting Information Tables S2 to S5).

**Table 3:** Comparison of the rs144051132 (T > A), rs186727643 (C > T), and rs188099931 (A > G) genotype and minor allele distributions between the control (CON) and anterior cruciate ligament (ACL) groups, as well as between the CON group and a subset of the ACL group (ACL‐NC), comprising those who sustained ACL ruptures via a non‐contact mechanism, of the combined cohorts (Australian, Polish, South African, and Swedish) of self‐reported European ancestry.

	**CON**	**ACL**	***p*‐value**	**ACL‐NC**	***p*‐value**
rs144051132	*n* = 568	*n* = 799		*n* = 441	
TT Genotype	99.3 (564)	99.0 (791)	0.770^a^ 0.557^b^^,^ d 0.886^c^^,^ d	99.1 (437)	0.735^a^ 0.719^b^^,^ g 0.930^c^^,^ g
TA Genotype	0.7 (4)	1.0 (8)		0.9 (4)	
A Allele	0.4 (4)	0.5 (8)	0.771^a^	0.5 (4)	0.735^a^
rs186727643	*n* = 555	*n* = 797		*n* = 440	
CC Genotype	100.0 (555)	99.9 (796)	1.000^a^ 1.000^b^^,^ e 0.427^c^^,^ e	99.8 (439)	0.442^a^ 0.442^b^^,^ e 0.271^c^^,^ e
CT Genotype	0.0 (0)	0.1 (1)		0.2 (1)	
T Allele	0.0 (0)	< 0.1 (1)	1.000^a^	0.1 (1)	0.442^a^
rs188099931	*n* = 574	*n* = 804		*n* = 447	
AA Genotype	99.8 (573)	99.4 (799)	0.410^a^ 0.187^b^^,^ f 0.360^c^^,^ f	99.3 (444)	0.324^a^ 0.204^b^^,^ h 0.361^c^^,^ h
AG Genotype	0.2 (1)	0.6 (5)		0.7 (3)	
G allele	0.1 (1)	0.3 (5)	0.411^a^	0.3 (3)	0.325^a^

*Note:* Genotypes and minor alleles are reported as a relative percentage with the total number of each genotypes or alleles in parenthesis. The minor AA, TT, and GG genotypes were not detected and thus excluded from the analysis.

^a^ Fisher's exact test.

^b^ codominat model of inheritance (the dominant, recessive, overdominant, and log‐additive models of inheritance were not observed).

^c^ codominat model co‐varied for country of recruitment.

^d^ Un‐adjusted odds ratio (OR) = 1.4% and 95% confidence interval (CI): 0.4–4.8, adjusted OR = 1.1% and 95% CI: 0.3–3.8.

^e^ A reliable OR and 95% CI could not be calculated.

^f^ Un‐adjusted OR = 3.6% and 95% CI: 0.4–30.7, adjusted OR = 2.5% and 95% CI: 0.3–22.7.

^g^ Un‐adjusted OR = 1.3% and 95% CI: 0.3–5.2, adjusted OR = 1.1% and 95% CI: 0.3–4.3.

^h^ Un‐adjusted OR = 3.9% and 95% CI: 0.4–37.4, adjusted OR = 2.7% and 95% CI: 0.3–26.2.

The minor homozygous rs144051132 AA, rs186727643 TT, and rs188099931 GG genotypes were entirely absent from all four cohorts. The heterozygous genotypes of all three polymorphisms were rare with only twelve (0.9%) rs144051132 TA, one (< 0.1%) rs186727643 CT and six (0.4%) rs188099931 AG genotypes identified in the combined European cohorts (Table 3). None of the heterozygous genotypes were detected in the Swedish cohort, while rs186727643 CT was absent from both the Polish and South African cohorts, and rs188099931 AG was absent from the Polish cohort (Supporting Information Tables S2 to S5).

None of the participants in the European populations were heterozygous for more than one of the three polymorphisms, where 0.7% (*n* = 4), 1.7% (*n* = 14), and 1.8% (*n* = 8) of the CON group, ACL group and ACL‐NC sub‐group had either a rs144051132 TA, rs186727643 CT, or rs188099931 AG genotype, respectively. The remaining participants in each group had the major homozygous genotype at each of the three polymorphisms. There was no significant difference in the distribution of participants with at least one heterozygous genotype when the combined ACL and CON groups were compared (codominant inheritance model, unadjusted *p* = 0.080, odds ratio = 2.5, 95% confidence interval: 0.8–7.7; country of recruitment adjusted *p* = 0.255, odds ratio = 1.9, 95% confidence interval: 0.6–5.9). Similarly, there was no significant difference when the ACL‐NC sub‐group and CON group were compared (codominant inheritance model, unadjusted *p* = 0.108, odds ratio = 2.6, 95% confidence interval: 0.8–8.7, country of recruitment adjusted *p* = 0.244, odds ratio = 2.0, 95% confidence interval: 0.6–6.8).

Similarly, there were no significant differences in the genotype or allele distributions of the three polymorphisms between the CON and ACL groups, or between the CON group and ACL‐NC sub‐group, when the self‐reported South African mixed ancestry cohort was analyzed (Table 4). These differences remained non‐significant after covarying for age in the CON versus ACL analysis and for age and BMI in the CON versus ACL‐NC analysis. All three minor homozygous genotypes were entirely absent. Only three (1.5%) rs144051132 TA, eight (4.0%) rs186727643 CT and one (0.5%) rs188099931 AG genotypes were detected in the mixed ancestry cohort (Table 4). None of the participants were heterozygous for more than one of the three polymorphisms, where 4.8% (*n* = 5), 7.2% (*n* = 7) and 4.0% (*n* = 2) of the CON group, ACL group and ACL‐NC sub‐group had either a rs144051132 TA, rs186727643 CT or rs188099931 AG genotype, respectively. Again, the remaining participants in each group had the major homozygous genotype at each of the three polymorphisms. There was no significant difference in the distribution of participants with at least one heterozygous genotype when the ACL and CON groups were compared (co‐dominant inheritance model, unadjusted *p* = 0.461, odds ratio = 1.6, 95% confidence interval: 0.5–5.1; age adjusted *p* = 0.948, odds ratio = 1.1, 95% confidence interval: 0.3–4.1). Similarly, there was no significant difference when the ACL‐NC sub‐group and CON group were compared (co‐dominant inheritance model, unadjusted *p* = 0.829, odds ratio = 0.8, 95% confidence interval: 0.2–4.5, age and BMI adjusted *p* = 0.756, odds ratio = 1.3, 95% confidence interval: 0.2–8.0).

**Table 4:** Comparison of the rs144051132 (T > A), rs186727643 (C > T), and rs188099931 (A > G) genotype and minor allele distributions between the control (CON) and anterior cruciate ligament (ACL) groups, as well as between the CON group and a subset of the ACL group (ACL‐NC), comprising those who sustained ACL ruptures via a non‐contact mechanism, in the South African mixed ancestry cohort.

	CON	ACL	*p*‐value	ACL‐NC	*p*‐value
	*n* = 105	*n* = 97		*n* = 50	
rs144051132					
TT Genotype	99.0 (104)	97.9 (95)	0.609^a^ 0.512^b^^,^ d 0.462^c^^,^ d	98.0 (49)	0.543^a^ 0.601^b^^,^ g 0.602^c^^,^ g
TA Genotype	1.0 (1)	2.1 (2)		2.0 (1)	
A Allele	0.5 (1)	1.0 (2)	0.608^a^	1.0 (1)	0.542^a^
rs186727643					
CC Genotype	97.1 (102)	94.8 (92)	0.485^a^ 0.401^b^^,^ e 0.931^c^^,^ e	98.0 (49)	1.000^a^ 0.748^b^^,^ h 0.638^c^^,^ h
CT Genotype	2.9 (3)	5.2 (5)		2.0 (1)	
T Allele	1.4 (3)	2.6 (5)	0.489^a^	1.0 (1)	1.000^a^
rs188099931					
AA Genotype	99.0 (104)	100.0 (97)	1.000^a^ 1.000^b^^,^ f 0.282^c^^,^ f	100.0 (50)	1.000^a^ 1.000^b^^,^ f 0.344^c^^,^ f
AG Genotype	1.0 (1)	0.0 (0)		0.0 (0)	
G allele	0.5 (0)	0.0 (0)	1.000^a^	0.0 (0)	1.000^a^

*Note:* Genotypes and minor alleles are reported as a relative percentage with the total number of each genotypes or alleles in parenthesis. The minor AA, TT, and GG genotypes were not detected and thus excluded from the analysis.

^a^ Fisher's exact test.

^b^ codominat model of inheritance (the dominant, recessive, overdominant, and log‐additive models of inheritance were not observed).

^c^ codominat model adjusted for age and BMI.

^d^ Un‐adjusted odds ratio (OR) = 2.2% and 95% confidence interval (CI): 0.2–24.5, adjusted OR = 2.4% and 95% CI: 0.2–27.4.

^e^ Un‐adjusted OR = 1.9% and 95% CI: 0.4–8.0, adjusted OR = 0.9% and 95% CI: 0.1–5.9.

^f^ A reliable OR and 95% CI could not be calculated.

^g^ Un‐adjusted OR = 2.1% and 95% CI: 0.1–34.6, adjusted OR = 2.1% and 95% CI: 0.1–35.4.

^h^ Un‐adjusted OR = 0.7% and 95% CI: 0.1–6.8, adjusted OR = 1.9% and 95% CI: 0.2–24.4.

### 3.3 Bioinformatic Analysis

Several bioinformatics tools and databases were used to evalute the potential functional effects of each polymorphism (Supporting Information Table S12). No clinical information was reported on ClinVar for any of the three polymorphisms. The low CADD scores (< 10.0) of all three polymorphisms indicate that they are most likely benign or of minimal clinical significance. Similarly, the low FATHMM scores (< 0.5) indicate that they are most likely benign or neutral.

Potential functional effects on RNA were determined for the intronic rs186727643 and intergenic rs188099931 polymorphisms using S‐fold software, which returns predictions of the secondary RNA structures and the Gibbs free energy (∆G) changes for each allele. Minimal conformational changes, as well as minimal (for rs186727643) or no (rs188099931) changes in the free energy of the predicted structures were observed, indicating that the sequence variations are unlikely to affect the stability of the transcribed RNA and are unlikely to have downstream effects on RNA function (Supporting Information Figure S7). As rs144051132 is a regulatory region polymorphism, it is not transcribed into RNA and was thus excluded from the S‐fold analysis.

## 4 Discussion

The primary aim of this study was to independently investigate the association of rs144051132 (T > A), rs186727643 (C > T), and rs188099931 (A > G) with ACL rupture risk in multiple cohorts. The second aim was to independently confirm the clinical viability of using these polymorphisms as markers of ACL rupture risk.

No significant associations were found between the three polymorphisms and ACL rupture risk across the four independent cohorts of self‐reported European ancestry when analsed independently or in combination, irrespective of whether the entire injury group was analyzed or only the sub‐group comprising individuals with a non‐contact mechanism of injury. There were also no significant associations when the South African mixed ancestry cohort was analyzed. Furthermore, the minor rs144051132 AA, rs1867276432 TT, and rs188099931 GG genotypes were entirely absent from all five cohorts. The heterozygous genotypes were also absent or rare in all the cohorts included in this study. As the power calculations indicated that the study had sufficient power to detect the larger effect sizes reported in the original study for rs144051132 and rs1867276432, the present study was underpowered to detect the smaller effect size reported for rs188099931. Although covarying the combined European analysis for country of birth reduces potential population stratification, it does not completely eliminate the possibility of residual stratification, particularly because ancestry was self‐reported and genetic ancestry principal components were not included in the analysis.

A primary concern for the use of these polymorphisms as injury risk markers pertains to their observed allele frequencies. The genotype and allele frequencies reported in this study were similar to those reported on the NCBI public database (Supporting Information Table S13). All three minor alleles (rs144051132 A, rs186727643 T, and rs188099931 G alleles) are either extremely rare or entirely absent from the five main population groups listed on the NCBI public database. The rs186727643 T and rs188099931 G alleles are absent in both South and East Asian populations. The remaining populations have minor allele frequencies ranging from 0.1% to 1.1%. The only exception is rs186727643 where the frequency of the minor T allele is 9.5% in the African population [5]. Based on the published effect sizes, the sample size of the combined European ancestry cohort was large enough to detect statistically significant independent associations with rs144051132 and rs186727643, but not with rs188099931 [5].

Initial interest in the use of these three polymorphisms as markers of ACL injury risk arose from the novel findings of a GWAS using data from the Kaiser Permanente Research Board (KPRB) and the UK Biobank where significant independent associations between these polymorphisms and ACL and/or PCL injury risk were reported, using a genome‐wide significance threshold of *p* < 5 × 10^−8^ [5].

Although the reported minor rs144051132 A (odds ratio = 2.9), rs186727643 T (odds ratio = 4.5), and rs188099931 G (odds ratio = 2.6) alleles were significantly associated with increased injury risk, the case group was highly heterogenous, comprising of participants who had sustained injury to the ACL and/or PCL, and included new and recurrent ligament sprains of undisclosed mechanism and severity [5]. Based on ICD‐9, ICD‐10, Read V2 or Read V3 international diagnostic codes, approximately 75% of the ACL and PCL injury cases were diagnosed with a sprain of the knee cruciate ligament. Other diagnoses included (i) PCL sprain, (ii) old and other spontaneous disruption of the PCL, (iii) ACL sprain, (iv) old and other spontaneous disruption of the ACL, (v) partial ACL rupture, (vi) complete ACL rupture and (vii) ACL reconstruction. In addition, only individuals of European ancestry were included in the analysis to reduce any effects of population stratification [5]. Furthermore, an ealier GWAS had failed to identify any polymorphisms significantly associated with ACL injury risk, including these three polymorphisms [17]. Homogeneity of case groups is critically important in studies involving musculoskeletal soft tissue injuries, such as ACL ruptures, owing to the complexity and multifactorial nature of such injuries. Thus, it was necessary to confirm the association of these polymorphisms with ACL rupture in a homogenous, well‐defined case group.

Functionally, rs144051132 is a regulatory region polymorphism, occurring specifically within an enhancer element, that results in a T > A nucleotide change within a FOS transcription factor binding site. The polymorphism is located downstream of the *LOC105375247* and *INHBA* genes. *INHBA* encodes inhibin βA, a signaling molecule and growth/differentiation factor involved in osteoblastic cell growth during bone development and the regulation of activin activity via its receptor, betaglycan [18]. A possible mechanism of action by which this polymorphism could contribute to injury susceptibility is by interfering with FOS transcription factor binding, leading to altered *INHBA* expression, and thus variation in osteoblastic cell growth and activity [5]. *LOC105375247* is a putative long non‐coding RNA (lncRNA), still requiring functional annotation. Generally, lncRNAs are involved in the regulation of gene expression and may contribute to the abnormal tissue‐specific gene expression patterns observed in many tissue pathologies [19]. However, the effect of rs144051132, if any, on *LOC105375247* is unclear.

Intronic polymorphism rs186727843 maps to the annoted genomic locus *LOC101928387*, which is positioned upstream of the *AEBP2* gene, encoding adipocyte enhancer (AE)‐binding protein 2, a DNA‐binding transcriptional activator that may play a regulatory role in the migration and development of neural crest cells [20]. The rs188099931 intergenic polymorphism is located downstream of the annotated locus *LOC101927869* and upstream of *LOC105372751*. As both rs188099931 and rs186727643 are non‐coding polymorphisms whose functional effects are unknown, it is unclear as to the exact mechanisms by which these polymorphisms may potentially contribute to the modulation of ACL rupture risk.

Thus, investigating the genomic context of each polymorphism yielded little evidence to suggest a biological relevance or potential contribution to ACL physiology or pathophysiology. Furthermore, multiple bioinformatic tools were used in this study to predict the potential functional and/or pathogenic effect of each polymorphism. Given the results, there was a very low likelihood of any one exhibiting clinical significance and the overarching conclusion being that none of the three polymorphisms was likely to have a deleterious or disease‐causing effect.

Even if an association had been identified between the polymorphisms analyzed in this study and ACL rupture risk, rare polymorphisms with low minor allele frequencies have substantially reduced statistical power to detect weak genetic effects [21]. When assessing the reliability and predictive value of a genetic test, polymorphisms with high sensitivity and specificity are preferred, as they minimize false‐negative and false‐positive results, respectively. In contrast, polymorphisms with low sensitivity and/or specificity are considerably less reliable and should not be used for risk assessment or to inform preventative interventions [21]. Given the rarity of the minor alleles for rs144051132, rs186727643, and rs188099931, although specificity would likely be high, the sensitivity of the original associations is expected to be very low [5]. In spite of this, rare variants, including those that have not been independently verified in subsequent studies, have been included and marketed in DTC genetic tests as markers for several musculoskeletal injuries [3].

Investigating the genetic contribution to musculoskeletal soft tissue injuries, such as ACL ruptures, is highly complex [22]. Genetic variability is just one of the many factors modulating injury risk, and the genetic contribution is polygenic with multiple gene‐gene interactions modulating injury risk. An added layer of complexity arises as many of the intrinsic risk factors themselves are multifactorial, each with their own genetic component [2]. While the possibilities of genetic testing, and personalized medicine as a whole, are extremely exciting, it is important to avoid overstating or overinterpreting scientific results to accelerate the advancement of technologies, thereby leading to premature commercialization of such technologies.

In conclusion, rs144051132, rs186727643, and rs188099931 were not associated with ACL rupture in the studied populations. These results, together with the rarity of the alleles, do not support the clinical validity of these polymorphisms as markers of ACL rupture risk.

## Author Contributions

**Sample recruitment:** Paweł Cieszczyk, Krzysztof Ficek, Charlotte K Häger, Eva‐Lena Stattintattin, Kjell G Nilsson, Julian A Feller, Oren Tirosh, Alison V September, Malcolm Collins. **Genotyping:** Caryn Phipson. **Analysis and drafting initial manuscript:** Caryn Phipson, Mary‐Jessica N. Laguette, Malcolm Collins. All authours reviewd and approved the final article.

## Conflicts of Interest

The authors declare no conflicts of interest.

## Supporting information


Supporting File 1

Supporting File 2

## Data Availability

The data that support the findings of this study are available from the corresponding author upon reasonable request.
