# Supplementary material for: Investigating the Association of Genetic Markers, Previously Identified by Genome‐Wide Association Studies, With ACL Rupture in Multiple Cohorts
Source: J Orthop Res. 2026 Sep 21;44(9):e70282. doi: 10.1002/jor.70282 (PMC13591651; doi:10.1002/jor.70282)
Supplement: Supplementary file 1 [file JOR-44-0-s001.docx]

**Supplementary Figure S1:** A typical scatter plot of the VIC- and FAM-labelled alleles providing a visual depiction of the genotype calling of rs144051132 (T>A). The relative accumulation of VIC and FAM fluorescence determines the plotted position of each sample. Red data points are homozygous for the VIC-labelled allele, blue data points are homozygous for the FAM-labelled allele, and green data points are heterozygous. VIC and FAM dye detect the T and A alleles, respectively, and so red data points are TT, blue data points are AA, and green data points are TA. Orange data points indicate no amplification. The synthesized oligomer probes used as positive test (PT) controls are labelled as reference points.

**Supplementary Figure S2:** A typical scatter plot of the VIC- and FAM-labelled alleles provides a visual depiction of the genotype calling for rs186727643 (C>T). The relative accumulation of VIC and FAM fluorescence determines the plotted position of each sample. Red data points are homozygous for the VIC-labelled allele, blue data points are homozygous for the FAM-labelled allele, and green data points are heterozygous. VIC and FAM dye detect the C and T alleles, respectively, and so red data points are CC, blue data points are TT, and green data points are CT. Orange data points indicate no amplification. The synthesized oligomer probes used as positive test (PT) controls are labelled as reference points.

**Supplementary Figure S3:** A typical scatter plot of the VIC- and FAM-labelled alleles provides a visual depiction of the genotype calling for rs188099931 (A>G). The relative accumulation of VIC and FAM fluorescence determines the plotted position of each sample. Red data points are homozygous for the VIC-labelled allele, blue data points are homozygous for the FAM-labelled allele, and green data points are heterozygous. VIC dye detects the A allele and so red data points are AA, blue data points are GG, and green data points are AG. Orange data points indicate no amplification. The synthesized oligomer probes used as positive test (PT) controls are labelled as reference points. The oligomer representing the homozygous GG positive control did not amplify and so there was no positive test control to use as a reference point for any samples assigned a GG genotype. All samples that were genotyped as a possible AG or GG, as well as a selection of samples that were genotyped as AA were sequenced.

**Supplementary Figure S4:** Typical chromatogram plots visualising the rs144051132 (T>A) sequencing data generated from Sanger sequencing. The reverse strand of each sample was sequenced and so the complementary alleles are shown in the plots. (**A)** This participant has the rs144051132 TT genotype: the A indicates homozygosity for the A allele on the reverse strand which means the forward strand will be homozygous for the T allele. (**B)** This participant has the rs144051132 TA genotype: the W indicates AT heterozygosity on the reverse strand and thus the forward strand will have a complementary K indicating TA heterozygosity.

**Supplementary Figure S5:** Typical chromatogram plots visualising the rs186727643 (C>T) sequencing data generated from Sanger sequencing. The reverse strand of each sample was sequenced and so the complementary alleles are shown in the plots. (**A)** This participant has the rs186727643 CC genotype: the G indicates homozygosity for the G allele on the reverse strand which means the forward strand will be homozygous for the C allele. (**B)** This participant has the rs186727643 CT genotype: the R indicates GA heterozygosity on the reverse strand and thus the forward strand will have a complementary Y indicating CT heterozygosity.

**Supplementary Figure S6:** Typical chromatogram plots visualising the rs188099931 (A>G) sequencing data generated from Sanger sequencing. The reverse strand of each sample was sequenced and so the complementary alleles are shown in the plots. (**A)** This participant has the rs188099931 AA genotype: the T indicates homozygosity for the T allele on the reverse strand which means the forward strand will be homozygous for the A allele. (**B)** This participant has the rs188099931 AG genotype: the Y indicates TC heterozygosity on the reverse strand and thus the forward strand will have a complementary R indicating AG heterozygosity.

**B**

**A**

**Supplementary Figure S7:** A comparison of the most stable predicted RNA secondary structures and Gibbs free energy change (∆G) of the **A)** rs186727643 C and T variants and **B)** rs188099931 A and G variants. The structures were generated using the S-fold online RNA folding tool (available at <https://sfold.wadsworth.org/cgi-bin/srna.pl>). The base changes can be observed at position 101 on each diagram. For rs186727643 T, the T has been replaced by a U as thymine is replaced by uracil when DNA is transcribed to RNA.
