# Supplementary material for: Investigating the Association of Genetic Markers, Previously Identified by Genome‐Wide Association Studies, With ACL Rupture in Multiple Cohorts
Source: J Orthop Res. 2026 Sep 21;44(9):e70282. doi: 10.1002/jor.70282 (PMC13591651; doi:10.1002/jor.70282)
Supplement: Supplementary file 2 [file JOR-44-0-s002.docx]

**Supplementary Table S1:** The sequences of the oligonucleotide probes synthesised for use as positive genotyping controls.

| **Variant allele** | **Synthesised oligonucleotide probe sequence** |
| --- | --- |
| **rs188099931** |  |
| **A** | 5’- ACC GAG TTG CCC GTT AAA GTG GAA GAA GGG AAA GAG GAA AAA AGG ACT TAA GTT TAT CTT AAA CAA GAT AG**A** CTT GTT TAA GGC TTG CTG TAT ATC TTA AAC AAA ATG CTA GTT GTA TAT TGT TTG CTT TAT AAA TCA CAT ACC CAT GTA GAG TTC GG -3’ |
| **G** | 5’- ACC GAG TTG CCC GTT AAA GTG GAA GAA GGG AAA GAG GAA AAA AGG ACT TAA GTT TAT CTT AAA CAA GAT AG**G** CTT GTT TAA GGC TTG CTG TAT ATC TTA AAC AAA ATG CTA GTT GTA TAT TGT TTG CTT TAT AAA TCA CAT ACC CAT GTA GAG TTC GG -3’ |
| **rs186727643** |  |
| **C** | 5’- ACC GAG TTG CCC GTT AAA GTC AGC ATT TAT TTC TTA TAT CTG GGA TTT TTT TCT TTC TTT CTT TTT TTT TTT TTT TTT TTG AGA CGG AGT CTC GTT **C**TG TCA CCC AGG CTG GAG TGC AGT GGC ATG ATC TCG GCT CAC TGC ACA TAC CCA TGT AGA GTT CGG -3’ |
| **T** | 5’- ACC GAG TTG CCC GTT AAA GTC AGC ATT TAT TTC TTA TAT CTG GGA TTT TTT TCT TTC TTT CTT TTT TTT TTT TTT TTT TTG AGA CGG AGT CTC GTT **T**TG TCA CCC AGG CTG GAG TGC AGT GGC ATG ATC TCG GCT CAC TGC ACA TAC CCA TGT AGA GTT CGG -3’ |
| **rs144051132** |  |
| **T** | 5’- AAT AGT ATG CAC ATG TAC TAG TTG GTG ACA GTC TAC TCC TGG GCC ACC AGT AGC AAG GGA TGA GAG TGA GAG GCA GAG CAG AGC CCT GGC **T**GA GTC ATC AGG ACA ACA GAC TAG CCT GTT TAT CAA ACC TTG CCA GAC AAG CAA AGG CTA ACT GAG AAG ATG GTT TTG TAA CTT GAG AAA GGG TTG TTT TG -3’ |
| **A** | 5’- AAT AGT ATG CAC ATG TAC TAG TTG GTG ACA GTC TAC TCC TGG GCC ACC AGT AGC AAG GGA TGA GAG TGA GAG GCA GAG CAG AGC CCT GGC **A**GA GTC ATC AGG ACA ACA GAC TAG CCT GTT TAT CAA ACC TTG CCA GAC AAG CAA AGG CTA ACT GAG AAG ATG GTT TTG TAA CTT GAG AAA GGG TTG TTT TG -3’ |

The polymorphic nucleotides are shown in bold and red.

**Supplementary Table S2:** Participant characteristics, as well as the relative rs144051132 (T>A), rs186727643 (C>T) and rs188099931 (A>G) genotype distributions, of the apparently healthy control (CON) and anterior cruciate ligament (ACL) groups, as well as between the CON group and a subset of the ACL group (ACL-NC), comprising those who sustained ACL ruptures via a non-contact mechanism, in the Australian cohort.

|  | **CON** | **ACL** | **p-value** | **ACL-NC** | **p-value** |
| --- | --- | --- | --- | --- | --- |
|  | n = 82 | n = 330 |  | n = 152 |  |
| **Characteristics:** |  |  |  |  |  |
| **Age** (years) | 31.0 (25.0; 39.0) ^1^ | 24.5 (18.0; 30.0) ^2^ | **<0.001** | 26.0 (19.0; 30.0) ^2^ | **<0.001** |
| **Sex** (% males) | 100.0 | 54.3 ^4^ | **<0.001** | 50.7 | **<0.001** |
| **Height** (cm) | 179.0 (175.0; 184.0) | 175.5 (167.3; 182.5) | **0.042** ^3^ | 175.8 (168.6; 182.0) | **0.016** ^3^ |
| **Weight** (kg) | 79.7 (72.9; 88.8) ^1^ | 76.3 (66.7; 85.6) ^2^ | 0.082 ^3^ | 76.6 (67.0; 88.0) ^2^ | **0.024** ^3^ |
| **BMI** (kg/m^2^) | 24.5 (22.9; 27.1) ^1^ | 24.6 (22.8; 27.3) ^2^ | 0.256 ^3^ | 25.0 (22.8; 27.8) ^2^ | 0.094 ^3^ |
| **rs144051132 (T>A):** |  |  |  |  |  |
| **TT Genotype** | 98.7 (79) | 98.5 (322) | 1.000 ^4^  0.859 ^5^  0.738 ^6^ | 98.7 (151) | 1.000 ^4^  0.970 ^5^  0.236 ^7^ |
| **TA Genotype** | 1.3 (1) | 1.5 (5) |  | 1.3 (2) |  |
| **rs186727643 (C>T):** |  |  |  |  |  |
| **CC Genotype** | 100.0 (77) | 99.7 (325) | 1.000 ^4^  1.000 ^5^  0.382 ^6^ | 93.3 (150) | 1.000 ^4^  1.000 ^5^  0.265 ^7^ |
| **CT Genotype** | 0.0 (0) | 0.3 (1) |  | 0.7 (1) |  |
| **rs188099931 (A>G):** |  |  |  |  |  |
| **AA Genotype** | 98.8 (81) | 98.8 (325) | 1.000 ^4^  0.998 ^5^  0.554 ^6^ | 98.0 (149) | 1.000 ^4^  0.663 ^5^  0.441 **^7^** |
| **AG Genotype** | 1.2 (1) | 1.2 (4) |  | 2.0 (3) |  |

The non-parametric continuous variables are expressed as medians and interquartile ranges (Q1; Q3), while categorical variables are expressed as percentages, with the n value for the genotypes in parathesis

p-values in bold indicate significance (p < 0.05)

None of the participants had the rare homozygous genotypes

BMI, body mass index; cm, centimetres; kg, kilograms; m, metres

^1^ Self-reported at time of recruitment, ^2^ self-reported at time of initial ACL injury, ^3^ since the CON group were all males, significant differences were only determined for the male sub-group only the male sub-group, medians and interquartile ranges are presented for all participants

^4^ Fisher’s exact test, ^5^ codominat model of inheritance (the dominant, recessive, overdominant, and log-additive models of inheritance were not observed), ^6^ codominat model co-varied for age, sex and height, ^7^ co-dominat model co-varied for age, sex, height and weight

Age (n): CON = 81; ACL = 265

Sex, Height, Weight and BMI (n): ACL = 265

**Supplementary Table S3:** Participant characteristics, as well as the relative rs144051132 (T>A), rs186727643 (C>T) and rs188099931 (A>G) genotype distributions, of the apparently healthy control (CON) and anterior cruciate ligament (ACL) groups, as well as between the CON group and a subset of the ACL group (ACL-NC), comprising those who sustained ACL ruptures via a non-contact mechanism, in the Polish cohort.

|  | **CON** | **ACL** | **p-value** | **ACL-NC** | **p-value** |
| --- | --- | --- | --- | --- | --- |
|  | n = 147 | n = 142 |  | n = 56 |  |
| **Characteristics:** |  |  |  |  |  |
| **Age** (years) | 20.4 (20.0; 22.5) ^1^ | 31.0 (23.0; 38.0) ^2^ | **<0.001** | 28.0 (22.0; 35.8) ^2^ | **<0.001** |
| **Sex** (% males) | 74.8 | 71.8 | 0.596 | 80.4 | 0.464 |
| **Height** (cm) | 178.1 ± 9.7 | 177.3 ± 9.6 | 0.491 | 178.2 ± 9.7 | 0.911 |
| **Weight** (kg) | 73.0 (64.4; 80.0) ^1^ | 78.0 (68.1; 87.0) ^1^ | 0.072 ^3^ | 78.5 (71.0; 85.0) ^1^ | 0.067 ^3^ |
| **BMI** (kg/m^2^) | 22.7 (21.2; 24.3) ^1^ | 24.9 (22.8; 26.7) ^1^ | 0.094 ^3^ | 24.6 (23.1; 25.8) ^1^ | 0.303 ^3^ |
| **rs144051132 (T>A):** |  |  |  |  |  |
| **TT Genotype** | 98.0 (144) | 99.3 (141) | 0.623 ^4^  0.319 ^5^  0.446 ^6^ | 98.2 (55) | 1.000 ^4^  0.906 ^5^  0.805 ^6^ |
| **TA Genotype** | 2.0 (3) | 0.7 (1) |  | 1.8 (1) |  |
| **rs186727643 (C>T):** |  |  |  |  |  |
| **CC Genotype** | 100.0 (136) | 100.0 (141) | 1.000 ^4,7^ | 100.0 (55) | 1.000 ^4,7^ |
| **CT Genotype** | 0.0 (0) | 0.0 (0) |  | 0.0 (0) |  |
| **rs188099931 (A>G):** |  |  |  |  |  |
| **AA Genotype** | 100.0 (147) | 100.0 (140) | 1.000 ^4,7^ | 100.0 (56) | 1.000 ^4,7^ |
| **AG Genotype** | 0.0 (0) | 0.0 (0) |  | 0.0 (0) |  |

The parametric and non-parametric continuous variables are expressed as medians and interquartile ranges (Q1; Q3) and mean ± standard deviation, respectively, while categorical variables are expressed as percentages, with the n value for the genotypes in parathesis

p-values in bold indicate significance (p < 0.05)

None of the participants had the rare homozygous genotypes

BMI, body mass index; cm, centimetres; kg, kilograms; m, metres

^1^ Self-reported at time of recruitment, ^2^ self-reported at time of initial ACL injury, ^3^ co-varied for age

^4^ Fisher’s exact test, ^5^ codominat model of inheritance (the dominant, recessive, overdominant, and log-additive models of inheritance were not observed), ^6^ codominat model co-varied for age, ^7^ monomorphic

Height (n): ACL = 139

Weight (n): ACL = 140

BMI (n): ACL = 137

**Supplementary Table S4:** Participant characteristics, as well as the relative rs144051132 (T>A), rs186727643 (C>T) and rs188099931 (A>G) genotype distributions, of the apparently healthy control (CON) and anterior cruciate ligament (ACL) groups, as well as between the CON group and a subset of the ACL group (ACL-NC), comprising those who sustained ACL ruptures via a non-contact mechanism, in the South African cohort of self-reported European ancestry.

|  | **CON** | **ACL** | **p-value** | **ACL-NC** | **p-value** |
| --- | --- | --- | --- | --- | --- |
|  | n = 229 | n = 242 |  | n = 161 |  |
| **Characteristics:** |  |  |  |  |  |
| **Age** (years) | 25.0 (22.0; 34.5) ^1^ | 22.0 (18.0; 32.0) ^2^ | M: **< 0.001**  F: **0.009** | 23.0 (19.0; 34.0) ^2^ | M: **0.009**  F: **0.026** |
|  |  | 27.0 (21.0; 41.0) ^1^ | 0.543 | 27.0 (21.1; 41.3) ^1^ | 0.451 |
| **Sex** (% males) | 56.3 | 74.0 | **<0.001** | 71.4 | **0.002** |
| **Height** (cm) | 175.0 (167.0; 181.0) | 179.0 (172.0; 185.0) | M: 0.201  F: 0.946 | 178.0 (170.7; 184.5) | M: 0.202  F: 0.808 |
| **Weight** (kg) | 73.0 (62.9; 80.6) ^1^ | 80.0 (69.0; 90.5) ^2^ | M: **0.003** ^3^  F: 1.000 | 80.0 (67.9; 90.0) ^2^ | M: **0.002** ^3^  F: 0.465 |
|  |  | 81.0 (70.0; 93.0) ^1^ | M: **< 0.001**  F: 0.400 | 80.0 (68.0; 90.0) ^1^ | M: **< 0.001**  F: 0.891 |
| **BMI** (kg/m^2^) | 23.5 (21.7; 25.5) ^1^ | 24.8 (22.5; 27.5) ^2^ | M: **0.020** ^3^  F: 0.735 | 25.7 (22.1; 27.1) ^2^ | M: **0.016** ^3^  F: 0.462 |
|  |  | 25.4 (23.0; 28.4) ^1^ | M: **< 0.001**  F: 0.682 | 25.1 (22.5; 28.0) ^1^ | M: **< 0.001**  F: 0.868 |
| **rs144051132 (T>A):** |  |  |  |  |  |
| **TT Genotype** | 100.0 (227) | 99.1 (233) | 0.499 ^4^  0.499 ^5^  0.160 ^6^ | 99.4 (154) | 0.406 ^4^  0.406 ^5^  0.225 ^6^ |
| **TA Genotype** | 0.0 (0) | 0.9 (2) |  | 0.6 (1) |  |
| **rs186727643 (C>T):** |  |  |  |  |  |
| **CC Genotype** | 100.0 (227) | 100.0 (236) | 1.000 ^4,7^ | 100.0 (156) | 1.000 ^4,7^ |
| **CT Genotype** | 0.0 (0) | 0.0 (0) |  | 0.0 (0) |  |
| **rs188099931 (A>G):** |  |  |  |  |  |
| **AA Genotype** | 100.0 (229) | 99.6 (239) | 1.000 ^4^  1.000 ^5^  0.183 ^6^ | 100.0 (160) | 1.000 ^4,7^ |
| **AG Genotype** | 0.0 (0) | 0.4 (1) |  | 0.0 (0) |  |

The non-parametric continuous variables are expressed as medians and interquartile ranges (Q1; Q3), while categorical variables are expressed as percentages, with the n value for the genotypes in parathesis

p-values in bold indicate significance (p < 0.05)

None of the participants had the rare homozygous genotypes

BMI, body mass index; cm, centimetres; kg, kilograms; m, metres; M, male; F, female

^1^ Self-reported at time of recruitment, ^2^ self-reported at time of initial ACL injury, ^3^ co-varied for age at injury for the ACL group, as well as the NON sub-group, and age at recruitment for the CON group

^4^ Fisher’s exact test, ^5^ codominat model of inheritance (the dominant, recessive, overdominant, and log-additive models of inheritance were not observed), ^6^ codominat model co-varied for age, sex and BMI, ^7^ monomorphic

Age (n): CON = 225; ACL ^2^ = 215; ACL ^1^ = 218; NON ^2^ = 155; NON ^1^ = 153

Height (n): CON = 221; ACL = 221; NON = 156

Weight (n): CON = 224; ACL ^2^ = 217; ACL ^1^ = 223; NON ^2^ = 154; NON ^1^ = 159

BMI (n): CON = 219; ACL ^2^ = 218; ACL ^1^ = 221; NON ^2^ = 154; NON ^1^ = 156

**Supplementary Table S5:** Participant characteristics, as well as the relative rs144051132 (T>A), rs186727643 (C>T) and rs188099931 (A>G) genotype distributions, of the apparently healthy control (CON) and anterior cruciate ligament (ACL) groups, as well as between the CON group and a subset of the ACL group (ACL-NC), comprising those who sustained ACL ruptures via a non-contact mechanism, in the Swedish cohort.

|  | **CON** | **ACL** | **p-value** | **ACL-NC** | **p-value** |
| --- | --- | --- | --- | --- | --- |
|  | n = 116 | n = 95 |  | n = 79 |  |
| **Characteristics:** |  |  |  |  |  |
| **Age** (years) | 46.0 (35.0; 54.3) ^1^ | 35.0 (25.0; 49.0) ^2^ | **<0.001** | 32.5 (24.0; 49.0) ^2^ | **<0.001** |
| **Sex** (% males) | 34.5 | 47.4 | 0.067 | 53.2 | **0.012** |
| **Height** (cm) | 171.0 (165.0; 179.8) | 174.0 (166.5; 179.0) | 0.421 | 174.0 (168.0; 179.0) | 0.209 |
| **Weight** (kg) | 72.0 (62.0; 80.0) ^1^ | 67.5 (59.3; 79.0) ^2^  75.0 (63.8; 84.0) ^1^ | 0.055  0.264 | 69.0 (60.0; 79.0) ^2^  75.0 (65.0; 84.0) ^1^ | 0.153  0.110 |
| **BMI** (kg/m^2^) | 24.3 (22.5; 26.1) ^1^ | 22.5 (21.0; 24.1) ^2^  24.5 (22.5; 26.5) ^1^ | **0.017** ^3^  0.571 | 22.6 (20.9; 24.3) ^2^  24.5 (22.7; 26.3) ^1^ | **0.032** ^3^  0.442 |
| **rs144051132 (T>A):** |  |  |  |  |  |
| **TT Genotype** | 100.0 (115) | 100.0 (95) | 1.000 ^4^ | 100.0 (79) | 1.000 ^4^ |
| **TA Genotype** | 0.0 (0) | 0.0 (0) |  | 0.0 (0) |  |
| **rs186727643 (C>T):** |  |  |  |  |  |
| **CC Genotype** | 100.0 (115) | 100.0 (94) | 1.000 ^4^ | 100.0 (78) | 1.000 ^4^ |
| **CT Genotype** | 0.0 (0) | 0.0 (0) |  | 0.0 (0) |  |
| **rs188099931 (A>G):** |  |  |  |  |  |
| **AA Genotype** | 100.0 (116) | 100.0 (95) | 1.000 ^4^ | 100.0 (79) | 1.000 ^4^ |
| **AG Genotype** | 0.0 (0) | 0.0 (0) |  | 0.0 (0) |  |

The non-parametric continuous variables are expressed as medians and interquartile ranges (Q1; Q3), while categorical variables are expressed as percentages, with the n value for the genotypes in parathesis

p-values in bold indicate significance (p < 0.05)

None of the participants had the rare homozygous genotypes

BMI, body mass index; cm, centimetres; kg, kilograms; m, metres

^1^ Self-reported at time of recruitment, ^2^ Self-reported at time of initial ACL injury, ^3^ Co-varied for age

^4^ Fisher’s exact test and momophorphic

Age (n): CON = 114; ACL = 93; NON = 78

Height (n): CON = 108; ACL = 85; NON = 71

Weight (n): CON = 107; ACL ^2^ = 76; ACL ^1^ = 86; NON ^2^ = 63; NON ^1^ = 71

BMI (n): CON = 107; ACL ^2^ = 73; ACL ^1^ = 84; NON ^2^ = 62; NON ^1^ = 70

**Supplementary Table S6:** The combined apparently healthy control (CON) and anterior cruciate ligament (ACL) groups physical characteristics of the rs144051132 (T>A) genotype groups of the combined Australian, Polish, South African and Swedish self-reported European ancestry cohorts.

|  | **TT** | **TA** | **p-value** |
| --- | --- | --- | --- |
|  | n = 1355 | n = 12 |  |
| **Age** (years) | 25.0 (21.0; 39.9), n = 767 ^1^  25.0 (21.0; 36.0), n = 1257 ^2^ | 23.5 (19.8; 31.3), n = 6 ^1^  23.5 (19.0; 28.3), n = 12 ^2^ | 0.449  0.131 |
| **Sex** (% males) | 62.8, n = 1304 | 75.0, n = 12 | 0.551 |
| **Height** (cm) | 176.0 (169.0; 183.0), n = 1243 | 176.5 (175.3; 179.5), n = 12 | 0.598 |
| **Weight** (kg) | 75.0 (66.0; 85.0), n = 990 ^1^  75.0 (65.0; 84.0), n = 1094 ^2^ | 79.9 (68.0; 86.5), n = 7 ^1^  76.0 (70.6; 82.5), n = 11 ^2^ | 0.586  0.598 |
| **BMI** (kg/m^2^) | 24.1 (21.6; 27.9), n = 978 ^1^  23.9 (22.2; 26.0), n = 1087 ^2^ | 25.5 (21.6; 27.9), n = 7 ^1^  25.5 (22.3; 26.4), n = 11 ^2^ | 0.754  0.636 |

Except for sex, which is expressed as a percentage, the remaining non-parametric continuous variables are expressed as medians and interquartile ranges (Q1; Q3)

None of the participants had the rare AA genotype

BMI, body mass index; cm, centimetres; kg, kilograms; m, metres

^1^ Age, weight and BMI of the ACL and CON participants at the time of recruitment

^2^ Age, weight and BMI of the ACL and CON participants at the time of injury and recruitment respectively

**Supplementary Table S7:** The combined apparently healthy control (CON) and anterior cruciate ligament (ACL) groups physical characteristics of the rs186727643 (C>T) genotype groups of the combined Australian, Polish, South African and Swedish self-reported European ancestry cohorts.

|  | **CC** | **CT** |
| --- | --- | --- |
|  | n = 1351 | n = 1 |
| **Age** (years) | 25.0 (21.0; 40.0), n = 761 ^1^  25.0 (21.0; 36.0), n = 1253 ^2^ | -  26.4 |
| **Sex** (% males) | 62.4 | 100.0 |
| **Height** (cm) | 176.0 (169.0; 183.0), n = 1239 | 180.0 |
| **Weight** (kg) | 75.2 (66.0; 85.0), n = 983 ^1^  75.0 (65.0; 84.0), n = 1090 ^2^ | -  82.0 |
| **BMI** (kg/m^2^) | 24.1 (22.3; 26.2), n = 971 ^1^  23.9 (22.2; 26.0), n = 1083 ^2^ | -  25.3 |

Except for sex, which is expressed as a percentage, the remaining non-parametric continuous variables of the major homozygous genotype are expressed as medians and interquartile ranges (Q1; Q3)

Age, height, weight and BMI of the single participant with a CT genotype is presented

None of the participants had the rare TT genotype

BMI, body mass index; cm, centimetres; kg, kilograms; m, metres

^1^ Age, weight and BMI of the ACL and CON participants at the time of recruitment

^2^ Age, weight and BMI of the ACL and CON participants at the time of injury and recruitment respectively

**Supplementary Table S8:** The combined apparently healthy control (CON) and anterior cruciate ligament (ACL) groups physical characteristics of the rs188099931 (A>G) genotype groups of the combined Australian, Polish, South African and Swedish self-reported European ancestry cohorts.

|  | **AA** | **AG** | **p-value** |
| --- | --- | --- | --- |
|  | n = 1372 | n = 6 |  |
| **Age** (years) | 25.0 (21.0; 40.0), n = 782 ^1^  25.0 (21.0; 36.0), n = 1273 ^2^ | 37.0 & 45.0, n = 2 ^1^  33.5 (18.5; 39.0), n = 6 ^2^ | n.d.  0.706 |
| **Sex** (% males) | 63.1, n = 1308 | 83.3, n = 6 | 0.423 |
| **Height** (cm) | 176.0 (169.0; 183.0), n = 1259 | 182.8 (180.3; 185.3), n = 6 | 0.051 |
| **Weight** (kg) | 75.1 (66.0; 85.0), n = 1004 ^1^  75.0 (65.0; 84.0), n = 1111 ^2^ | 78.2 & 80.0, n = 2 ^1^  79.1 (66.6; 91.3), n = 6 ^2^ | n.d.  0.537 |
| **BMI** (kg/m^2^) | 24.1 (22.3; 26.2), n = 992 ^1^  23.9 (22.2; 26.0), n = 1104 ^2^ | 23.4 & 24.2, n = 2 ^1^  23.8 (20.3; 25.9), n = 6 ^2^ | n.d.  0.605 |

Except for sex, which is expressed as a percentage, the remaining non-parametric continuous variables of the major homozygous, as well as some of the heterozygous genotypes are expressed as medians and interquartile ranges (Q1; Q3), the values of the remaining AG genotype variables are presented

None of the participants had the rare GG genotype

BMI, body mass index; cm, centimetres; kg, kilograms; m, metres; n.d., not determined

^1^ Age, weight and BMI of the ACL and CON participants at the time of recruitment

^2^ Age, weight and BMI of the ACL and CON participants at the time of injury and recruitment respectively

**Supplementary Table S9:** The combined apparently healthy control (CON) and anterior cruciate ligament (ACL) groups physical characteristics of the rs144051132 (T>A) genotype groups of South African mixed ancestry cohort at the time of recruitment.

|  | **TT** | **TA** |
| --- | --- | --- |
|  | n = 199 | n = 3 |
| **Age** (years) | 26.0 (22.0; 31.0), n = 194 | 24 to 30, n =3 |
| **Sex** (% males) | 81.9, n = 199 | 100.0, n = 3 |
| **Height** (cm) | 172.8 ± 8.5, n = 184 | 170.0 and 175.0, n = 2 |
| **Weight** (kg) | 76.0 (66.2; 88.0), n = 186 | 65.0 to 89.0, n = 3 |
| **BMI** (kg/m^2^) | 25.7 (22.7; 28.1), n = 182 | 21.2 and 26.0, n = 2 |

Except for sex, which is expressed as a percentage, the remaining continuous variables for the major homogeneous TT genotype are expressed as average ± standard deviation or median and interquartile range (Q1; Q3) for parametric and non-parametric data respectively

Age, height, weight and BMI of the three participants with a TA genotype is presented as the range

None of the participants had the rare AA genotype

BMI, body mass index; cm, centimetres; kg, kilograms; m, metres

**Supplementary Table S10:** The combined apparently healthy control (CON) and anterior cruciate ligament (ACL) groups physical characteristics of the rs186727643 (C>T) genotype groups of South African mixed ancestry cohort at the time of recruitment.

|  | **CC** | **CT** | **p-value** |
| --- | --- | --- | --- |
|  | n = 194 | n = 8 |  |
| **Age** (years) | 26.0 (22.0; 31.0), n = 190 | 27.0 (24.0; 33.0), n = 7 | 0.457 |
| **Sex** (% males) | 82.0, n = 194 | 87.5, n = 8 | 1.000 |
| **Height** (cm) | 172.7 ± 8.4, n = 180 | 177.3 ± 9.4, n = 6 | 0.281 |
| **Weight** (kg) | 76.0 (66.0; 88.0), n = 183 | 75.0 (68.4; 101.3), n = 6 | 0.649 |
| **BMI** (kg/m^2^) | 25.7 (22.7; 28.1), n = 178 | 25.3 (23.4; 28.4), n = 6 | 0.752 |

Except for sex, which is expressed as a percentage, the remaining continuous variables are expressed as average ± standard deviation or median and interquartile range (Q1; Q3) for parametric and non-parametric data respectively

None of the participants had the rare TT genotype

BMI, body mass index; cm, centimetres; kg, kilograms; m, metres

**Supplementary Table S11:** The combined apparently healthy control (CON) and anterior cruciate ligament (ACL) groups physical characteristics of the rs188099931 (A>G) genotype groups of South African mixed ancestry cohort at the time of recruitment.

|  | **AA** | **AG** |
| --- | --- | --- |
|  | n = 201 | n = 1 |
| **Age** (years) | 26.0 (22.0; 31.0), n = 196 | 28 |
| **Sex** (% males) | 82.6, n = 201 | 0.0 |
| **Height** (cm) | 172.9 ± 8.4, n = 185 | 155.0 |
| **Weight** (kg) | 76.0 (66.5; 88.0), n = 188 | 53.0 |
| **BMI** (kg/m^2^) | 25.8 (22.7; 28.1), n = 183 | 22.1 |

Except for sex, which is expressed as a percentage, the remaining continuous variables for the major homogeneous AA genotype are expressed as average ± standard deviation or median and interquartile range (Q1; Q3) for parametric and non-parametric data respectively

Age, height, weight and BMI of the single participant with a AG genotype is presented

None of the participants had the rare GG genotype

BMI, body mass index; cm, centimetres; kg, kilograms; m, metres

**Supplementary Table S12** Evaluation of the potential functional and/or pathogenic effects of each variant using different bioinformatics tools and databases, namely VEP, ClinVar, CADD, FATHMM, and S-fold.

| **Variant** | **Consequence (VEP)** | **ClinVar** | **CADD** | **FATHMM** | **S-fold** |
| --- | --- | --- | --- | --- | --- |
| **rs144051132** | Regulatory region variant | Not reported | 2.231 | 0.15098 | — |
| **rs186727643** | Intronic variant | Not reported | 0.136 | 0.04559 | Minimal change in ∆G |
| **rs188099931** | Intergenic variant | Not reported | 2.156 | 0.09192 | No change in ∆G |

**Supplementary Table S13:** Genotype and minor allele frequencies of the rs144051132 (T>A), rs186727643 (C>T), and rs188099931 (A>G) variants within the five main population groups listed on the NCBI public database, as well as the minor allele ranges in the sub-population groups (Sub-Pop).

| **Population** | **Major Homozygous**  **Genotype** (%) | | **Heterozygous**  **Genotype**  (%) | **Minor Allele**  (%) | **Heterozygous**  **Genotype**  **Sub-Pop Range**  (%) | | **Minor Allele Sub-Pop Range** (%) |
| --- | --- | --- | --- | --- | --- | --- | --- |
| **rs144051132 (T>A)** | | | | | | | |
| African (AFR) | 99.8 | | 0.2 | 0.1 | 0.0 - 1.6 | | 0.0 - 0.8 |
| European (EUR) | 97.8 | | 2.2 | 1.1 | 1.0 - 4.7 | | 0.5 - 2.3 |
| East Asian | 100.0 | | 0.0 | 0.0 | 0.0 | | 0.0 |
| South Asian (SAS) | 100.0 | | 0.0 | 0.0 | 0.0 | | 0.0 |
| American (AMR) | 100.0 | | 0.0 | 0.0 | 0.0 | | 0.0 |
| **rs186727643 (C>T)** | | | | | | | |
| African (AFR) | - | - | | 9.5 | - | - | |
| European (EUR) | - | - | | 0.2 | - | - | |
| East Asian | - | - | | 0.0 | - | - | |
| South Asian (SAS) | - | - | | 0.0 | - | - | |
| American (AMR) | - | - | | 1.6 | - | - | |
| **rs188099931 (A>G)** | | | | | | | |
| African (AFR) | 100.0 | 0.0 | | 0.0 | 0.0 | 0.0 | |
| European (EUR) | 99.8 | 0.2 | | 0.1 | 0.0 - 1.0 ^1^ | 0.0 - 0.5 | |
| East Asian | 100.0 | 0.0 | | 0.0 | 0.0 | 0.0 | |
| South Asian (SAS) | 100.0 | 0.0 | | 0.0 | 0.0 | 0.0 | |
| American (AMR) | 99.4 | 0.6 | | 0.3 | 0.0 - 1.1 | 0.0 - 0.5 | |

All genotype and allele frequencies, as well as the ranges within sub-populations, are reported as relative percentages and were obtained from NCBI: 1000 Genomes Project Phase 3 (accessed 16 January 2026: <https://www.ncbi.nlm.nih.gov>)

The major homozygous genotypes for rs144051132, rs186727643, and rs188099931 are TT, CC, and AA, respectively

The rare homogeneous genotype was not present for any of the three polymorphisms

The minor alleles for rs144051132, rs186727643, and rs188099931 are A, T, and G, respectively

American refers to Admixed/Latino ancestry

No genotype of sub-population data is available for rs186727643

^1^ Only present in the CEU (Utah residents with Northern and Western European ancestry) sub-population, absent in the FIN (Finish), GBR (British individuals from England and Scotland**)**, IBS (Spanish Iberian populations**)** and TSI (Italian Tuscans**)** sub-populations
